# Chlorpromazine, a Clinically Approved Drug, Inhibits SARS-CoV-2 Nucleocapsid-Mediated Induction of IL-6 in Human Monocytes

**DOI:** 10.3390/molecules27123651

**Published:** 2022-06-07

**Authors:** Iwona Karwaciak, Kaja Karaś, Anna Sałkowska, Joanna Pastwińska, Marcin Ratajewski

**Affiliations:** Laboratory of Epigenetics, Institute of Medical Biology, Polish Academy of Sciences, 93-232 Lodz, Poland; isachrajda@cbm.pan.pl (I.K.); kkaras@cbm.pan.pl (K.K.); asalkowska@cbm.pan.pl (A.S.); jpastwinska@cbm.pan.pl (J.P.)

**Keywords:** SARS-CoV-2, COVID-19, Interleukin 6, monocytes, chlorpromazine, cytokine storm

## Abstract

The COVID-19 pandemic, caused by the rapidly spreading SARS-CoV-2 virus, led to the unprecedented mobilization of scientists, resulting in the rapid development of vaccines and potential pharmaceuticals. Although COVID-19 symptoms are moderately severe in most people, in some cases the disease can result in pneumonia and acute respiratory failure as well as can be fatal. The severe course of COVID-19 is associated with a hyperinflammatory state called a cytokine storm. One of the key cytokines creating a proinflammatory environment is IL-6, which is secreted mainly by monocytes and macrophages. Therefore, this cytokine has become a target for some therapies that inhibit its biological action; however, these therapies are expensive, and their availability is limited in poorer countries. Thus, new cheaper drugs that can overcome the severe infections of COVID-19 are needed. Here, we show that chlorpromazine inhibits the expression and secretion of IL-6 by monocytes activated by SARS-CoV-2 virus nucleocapsid protein and affects the activity of NF-κB and MEK/ERK signaling. Our results, including others, indicate that chlorpromazine, which has been used for several decades as a neuroleptic, exerts antiviral and immunomodulatory activity, is safe and inexpensive, and might be a desirable drug to support the therapy of patients with COVID-19.

## 1. Introduction

The coronavirus disease 2019 (COVID-19) pandemic has led to more than 6 million deaths to date (May 2022) [1]. This disease is caused by the highly pathogenic and readily transferable SARS-CoV-2 virus [2] belonging to the family *Coronaviridae* and *genus Betacoronavirus* [3,4]. The disease is particularly dangerous for elderly patients and patients with chronic medical conditions who may develop severe COVID-19 [5]. Severe COVID-19 is characterized by hyperinflammation and cytokine storms, which leads to damage to tissues and organs and, as a consequence, can cause acute respiratory distress syndrome (ARDS) and death. In critically ill patients, high levels of IL-6, IL-1β, IP-10, MCP-1, and TNF-α were detected [6,7,8]. Among these cytokines, IL-6 seems to be particularly important, as very high levels of it have been associated with the severity of the disease [9], [10,11,12], making it an attractive target in anti-COVID-19 therapy [13]. One of the main sources of this cytokine are monocytes and macrophages that produce it in response to viral infection [14,15], and thus participate in the course of COVID-19 [16,17].

Previously, we found that the most immunogenic SARS-CoV-2 proteins, spike (S) and nucleocapsid (N), induce the expression and release of IL-6 in monocytes and macrophages [18], suggesting that the interaction of these cells by virus particles contributes significantly to the development of cytokine storms. Interestingly, in contrast to the activation of Th1 lymphocytes, where both proteins had similar effects [19], the nucleocapsid was much more efficient in inducing *IL6* expression in monocytes and macrophages [18]. This prompted us to search for a compound that could diminish SARS-CoV-2 nucleocapsid-mediated IL-6 release from monocytes. We found that chlorpromazine (CPZ) has the ability to inhibit the induction of *IL6* by SARS-CoV-2 proteins in a process that involves the NF-κB transcription factor. Our findings support those of others [20,21,22], and suggest that chlorpromazine is a potential therapeutic drug for COVID-19.

## 2. Results

**Figure 1:**
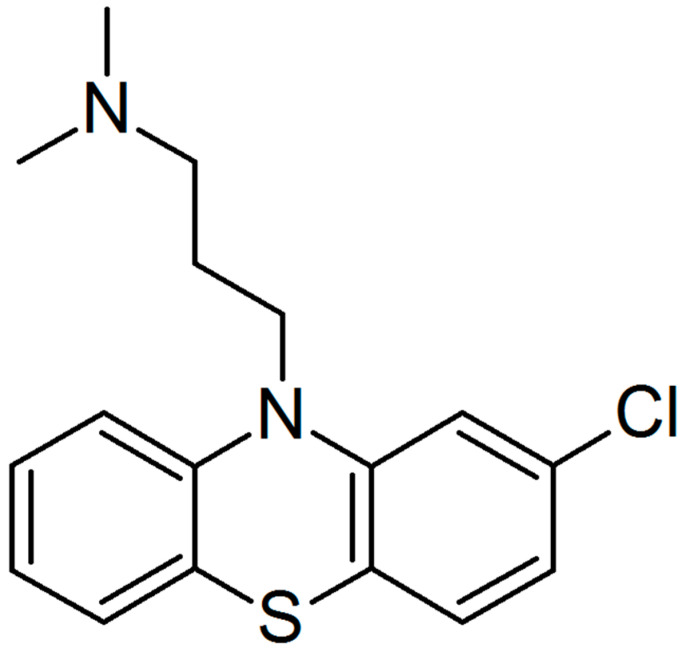
Structure of chlorpromazine.

**Figure 2:**
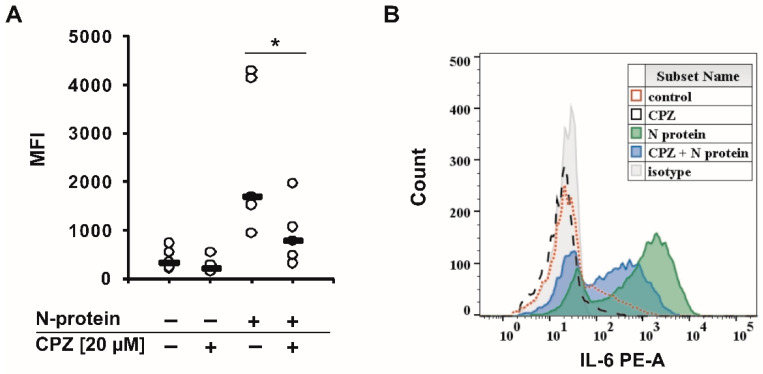
Chlorpromazine inhibits SARS-CoV-2 nucleocapsid-mediated induction of IL-6 protein expression in human monocytes isolated from healthy donors. Monocytes were pretreated with chlorpromazine (20 μM) for 3 h, treated with mock or SARS-CoV-2 nucleocapsid (1 μg/mL) for 2 h, stained with IL-6-PE or isotype control antibody and analyzed using flow cytometry. (**A**) Data (fluorescence intensity) are shown as dot plots from five donors (n = 5). Asterisks indicate a statistically significant difference at *p* < 0.05. Statistical analysis was performed using Friedman Repeated Measures ANOVA on Ranks followed by Student-Newman–Keuls post hoc test. (**B**) Representative histogram of intracellular IL-6.

**Figure 3:**
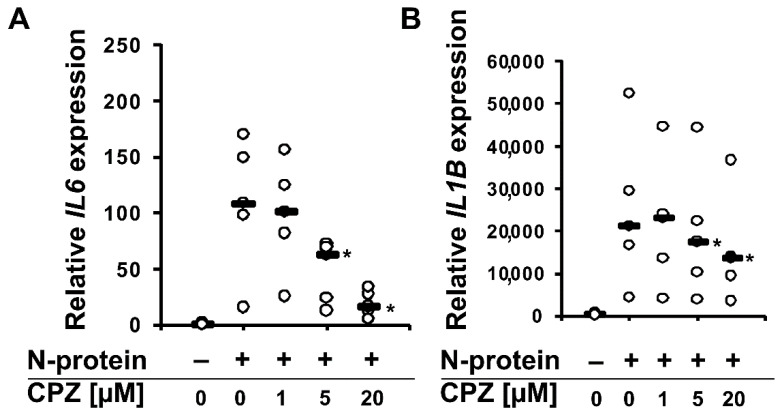
Chlorpromazine inhibits the SARS-CoV-2 nucleocapsid-mediated induction of *IL6* and *IL1B* mRNA expression in human monocytes isolated from healthy donors. Human monocytes were pretreated with the indicated chlorpromazine concentrations for 3 h, and after that time, the cells were stimulated with 1 μg/mL SARS-CoV-2 nucleocapsid for 48 h. Then, the cells were collected for RNA extraction. *IL6* (**A**) and *IL1B* (**B**) mRNA expression was determined by RT–PCR and normalized to averaged reference mRNA levels of the housekeeping genes *HPRT1*, *HMBS*, and *RPL13A*. Data are shown as dot plots with median values from five independent donors (n = 5). Asterisks indicate a statistically significant difference at *p* < 0.05. Statistical analysis was performed using Friedman Repeated Measures ANOVA on Ranks followed by Student-Newman–Keuls post hoc test.

**Figure 4:**
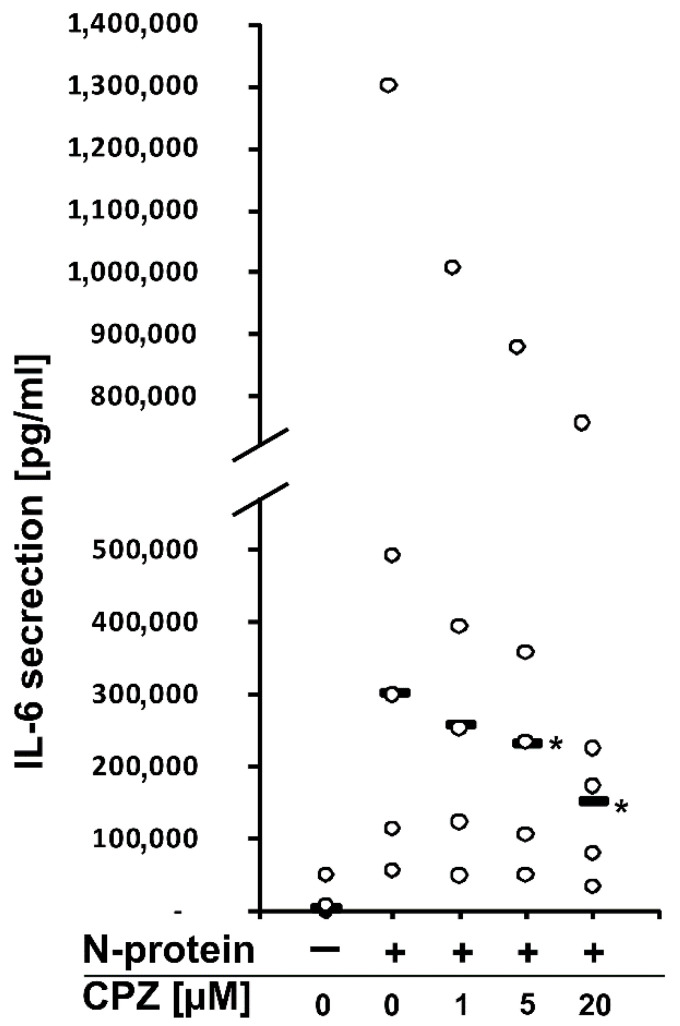
Chlorpromazine inhibits the SARS-CoV-2 nucleocapsid-mediated release of IL-6 in human monocytes isolated from healthy donors. Human monocytes were pretreated with the indicated chlorpromazine concentrations for 3 h; after pretreatment, the cells were stimulated with SARS-CoV-2 nucleocapsid protein at a concentration of 1 μg/mL for 48 h. Then, the cells were collected for RNA extraction, and the supernatants were collected for ELISA. IL-6 concentrations were determined using the human IL-6 Quantikine ELISA Kit. Data are shown as dot plots with median values from five independent donors (n = 5). Asterisks indicate a statistically significant difference at *p* < 0.05. Statistical analysis was performed using Friedman Repeated Measures ANOVA on Ranks followed by Student-Newman–Keuls post hoc test.

**Figure 5:**
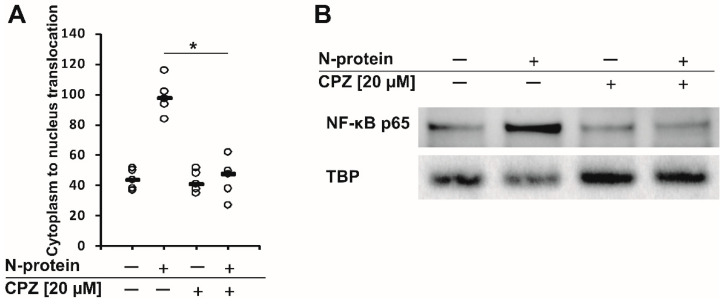
Chlorpromazine inhibits the translocation of the NF-κB transcription factor from the cytoplasm to the nucleus mediated by the SARS-CoV-2 nucleocapsid. (**A**) Human monocytes were pretreated with chlorpromazine (20 μM) for 3 h and then stimulated with 1 μg/mL SARS-CoV-2 nucleocapsid for 1 h. Cells were then stained with NF-κB p65 (D14E12) Alexa Fluor 488 conjugated antibody, and fluorescence was measured using the Cellomics ArrayScan HCS Reader. Data are shown as dot plots with median values from five independent donors (n = 5). Asterisks indicate a statistically significant difference at *p* < 0.05. Statistical analysis was performed using Friedman Repeated Measures ANOVA on Ranks followed by Student-Newman–Keuls post hoc test. (**B**) Western blotting results showing an increased level of NF-κB p65 in nuclear fractions after treatment with 1 μg/mL SARS-CoV-2 nucleocapsid and inhibition of translocation into the nucleus by chlorpromazine (20 μM, 3 h). TBP was used as a loading control.

**Figure 6:**
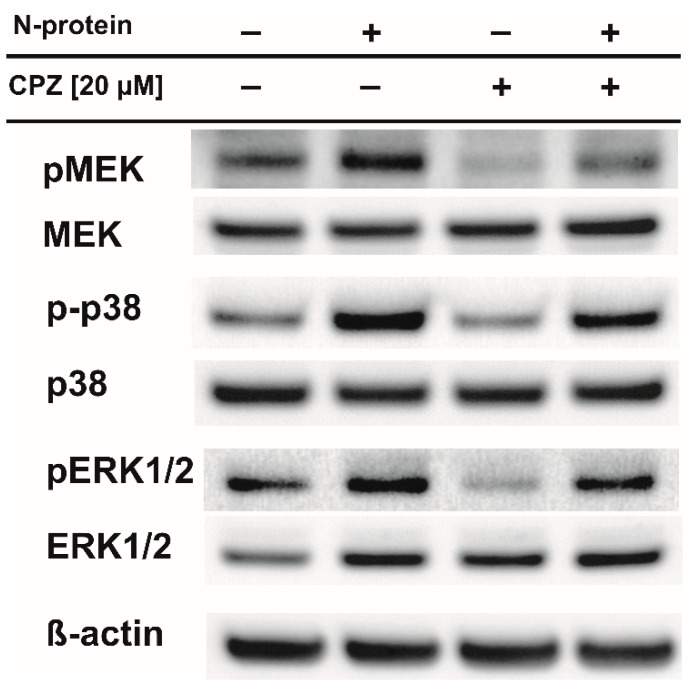
Chlorpromazine impairs cellular signaling via MEK/ERK in human monocytes induced by the nucleocapsid of SARS-CoV-2. Human monocytes and those pretreated with chlorpromazine (20 μM, 3 h) were treated with the SARS-CoV-2 nucleocapsid for 1 h (1 μg/mL). After that time, protein lysates were prepared and analyzed by Western blotting.

Recently, we found that the spike and nucleocapsid proteins of SARS-CoV-2 mediate the inflammatory process in human monocytes, and interestingly, the nucleocapsid is a stronger inducer of proinflammatory IL-6 and IL-1β than the spike protein [18]. Because IL-6 is implicated in the pathological conditions and severity of COVID-19 [16,17], we decided to perform a literature search to find an approved drug that would inhibit the expression/secretion of this interleukin and the number of monocytes and macrophages infiltrating the lungs. Such conditions were met by chlorpromazine [23,24], (Figure 1), which has been suggested to treat COVID-19 patients. In the initial approach, using flow cytometry, we analyzed the effectiveness of chlorpromazine in inhibiting the expression of IL-6 in monocytes stimulated with the nucleocapsid of the novel coronavirus. As shown in Figure 2, the expression of IL-6 was significantly induced just after 2 h of stimulation with nucleocapsid protein, and was substantially diminished in cells pretreated with chlorpromazine (Figure 2). In the next set of experiments, we analyzed the expression of *IL6* and *IL1B* in monocytes exposed to the nucleocapsid for a longer period of time (48 h) and observed very high induction of the expression of these genes (Figure 3), as described previously [18]. Interestingly, pretreatment of monocytes with chlorpromazine diminished nucleocapsid-induced *IL6* expression in a dose-dependent manner (Figure 3A) and *IL1B* (Figure 3B). These results were confirmed at the protein level using ELISA; however, it should be noted that the effect of chlorpromazine on IL-6 protein expression was slightly weaker than its effect on *IL6* mRNA expression (Figure 4). Numerous studies have indicated that the NF-κB transcription factor is essential in the process of activation of proinflammatory cytokines, including IL-6, by SARS-CoV and SARS-CoV-2 nucleocapsid proteins [18,25,26,27,28,29]. We thus examined how chlorpromazine affects the translocation of NF-κB into the nucleus after cell activation by the novel coronavirus nucleocapsid. As shown in Figure 5A, treatment of monocytes with the nucleocapsid caused increased accumulation of NF-κB in the nucleus and in nuclear extracts (Figure 5B and Figure S1), and pretreatment with chlorpromazine inhibited this process. It is well known that activation of the MEK/ERK pathway is important for the entry, viral transcription, particle production, and replication of several viruses [30,31,32,33,34], and, recently, a MEK1/2 inhibitor was shown to inhibit SARS-CoV-2 replication and to impair the production of proinflammatory cytokines [35]. Analysis of signaling pathways indicated that nucleocapsid induced the phosphorylation of MEK, p38, and ERK1/2, and this effect was diminished by the pretreatment of cells with chlorpromazine (Figure 6 and Figure S2).

## 3. Discussion

During the COVID-19 pandemic, more than 527 million people worldwide were infected with the SARS-CoV-2 coronavirus, resulting in more than 6.3 million deaths (May 2022 [1]). COVID-19 is mild to moderately severe in most people infected with the virus; however, in some patients, the disease is severe, resulting in a cytokine storm and pneumonia [36,37]. A cytokine storm is a condition in which the immune system responds inappropriately to a pathogen, leading to overactivation of immune cells and the secretion of extraordinary amounts of cytokines and chemokines. The activated cells, *via* the proteins they secrete, damage surrounding tissues and organs and can result in acute respiratory failure syndrome (ARDS), failure of other organs and death [38,39,40]. A cytokine storm is characterized by extremely high levels of proinflammatory cytokines, e.g., interferons, TNF-α, IL-1β, IL-6, and IL-8, and chemokines, e.g., IP-10, MCP-1, MIP-1, and MIG [41,42,43]. In COVID-19 patients, high levels of TNF-α, IL-1β, IL-4, IL-6, IL-10, IP-10, MCP-1, and MIP-1A have been detected [7,8,44], and particularly, high IL-6 concentrations are positively correlated with the severity of the disease [6,45]. Activated monocytes and monocyte-derived macrophages are considered a major source of IL-6, and high numbers of these cells are found in the lungs of patients with severe COVID-19 [46,47,48,49,50]. This overactivation of the immune system is why some therapeutic approaches against COVID-19 focus on pharmaceuticals that can reduce the proinflammatory effects of IL-6, such as tocilizumab and sarilumab, which are inhibitors of the IL-6 receptor and have been shown to be effective in reducing the severity of the course of this disease [51,52,53]. However, tocilizumab and sarilumab are expensive drugs, and thus, other, cheaper and more easily available options are needed to mitigate the effects of the disease, especially in third world countries where vaccination coverage is still very low.

In this work, we show that the antipsychotic drug chlorpromazine (Figure 1), which is a phenothiazine derivative containing an extra chlorine atom in one of the benzene rings and a dimethylaminopropyl group at the heterocyclic nitrogen atom [54], inhibits SARS-CoV-2 nucleocapsid-induced expression of *IL6* and its release from human monocytes (Figure 2, Figure 3 and Figure 4). Furthermore, this compound affects the translocation of the NF-κB transcription factor into the nucleus (Figure 5 and Figure S1), which is crucial for the regulation of the *IL6* gene in response to stimuli, such as bacterial LPS, cytokines, and viruses [55,56,57,58,59]. Analysis of cellular signaling revealed that some elements important for the immunological activation of monocytes, e.g., MAPK signaling [60,61,62], are also diminished in cells pretreated with chlorpromazine. This finding is in line with the results of other studies and suggests that chlorpromazine has immunomodulatory effects, e.g., decreasing the levels of the proinflammatory cytokines TNF-α, IL-1β, and IL-2 [63,64,65], and increasing IgM blood levels [66]. In murine macrophages, chlorpromazine has already been shown to inhibit LPS-mediated induction of IL-6 expression [23,67], and similarly, this compound diminished the toxic effects of IL-1β [68] and induced the expression of the anti-inflammatory cytokine IL-10 in the brain [69]. This suggests that chlorpromazine might be beneficial for patients suffering from the neurological symptoms of COVID-19 caused by neuroinflammation [70,71,72]. Importantly, in contrast to remdesivir and tocilizumab, drugs that are already used for COVID-19 treatment, chlorpromazine, due to its lipophilicity, crosses the blood–brain barrier [73], and its concentrations in the brain are up to 25-fold higher than those in plasma [74]. As mentioned above, chlorpromazine, in addition to being an antipsychotic medication, modulates immunological responses and has antiviral properties; it inhibits replication of influenza virus as well as the coronaviruses SARS-CoV-1 and MERS-CoV [75,76,77,78]. Recently, chlorpromazine was shown to also be active against SARS-CoV-2 [73,79,80].

After administration, chlorpromazine concentrations in plasma reach up to 1 μM [81]; thus, there are some doubts that this medication can be effective in antiviral therapy as the concentrations needed to inhibit viral replication are higher [72]. However, the distribution of chlorpromazine in several organs is different than that in plasma; for example, in the lungs, the concentrations of the compound can be 200-fold higher than those in plasma [82], and in saliva, the detected concentrations can be up to 69 μM [73,83]. These concentrations are within the ranges in which positive effects of chlorpromazine have been observed in in vitro studies against SARS-CoV-2. The high concentrations of chlorpromazine in the lungs or in the liver are likely related to the fact that these organs contain a large number of lysosomes [84,85], having an acidic environment, and it was demonstrated that compounds with pKa > 8 are characterized by preferential uptake by the lungs, liver or kidneys [86]. Thus, chlorpromazine, as a basic lipophilic drug with pKa = 9.2, accumulates in organs enriched in lysosomes in a process known as lysosomal trapping [87]. Furthermore, considering that monocytes and macrophages also have a significant number of lysosomes [88,89], it is expected that these cells will also be directly targeted by chlorpromazine. Additionally, Weston et al. [79] showed that treatment with chlorpromazine protects mice infected with SARS-CoV from signs of the disease. Another aspect of chlorpromazine to consider that supports its use as an anti-COVID-19 drug is that it is an inhibitor of clathrin-mediated endocytosis [90,91], which is essential for SARS-CoV-2 entry into cells [92]. The use of inhibitors of this process significantly reduces the infectivity of this virus [92].

## 4. Materials and Methods

### 4.1. Monocyte Isolation

Monocytes were isolated using the Classical Monocyte Isolation Kit, human 130-117-337 from Miltenyi Biotec (Bergisch Gladbach, Germany) from PBMCs obtained from buffy coats of healthy, anonymous donors. Buffy coats were purchased from the Regional Center for Blood Donation and Blood Treatment, Łódź, Poland, as waste material. The cells were cultured in RPMI 1640 medium containing 10% fetal bovine serum (PAN Biotech, Aidenbach, Germany) and 10% human AB serum (PAN Biotech).

### 4.2. Proteins and Chemicals

COVID-19 nucleocapsid protein (cat. no. 32-190001) was purchased from Abeomics (San Diego, CA, USA). Chlorpromazine (cat. no. 285374) was purchased from Merck (Darmstadt, Germany). The purity of chlorpromazine was 98%.

### 4.3. Intracellular IL-6 Staining

For intracellular IL-6 staining, monocytes were pretreated, where indicated, with chlorpromazine (20 μM) for 3 h and stimulated with nucleocapsid (1 μg/mL) in the presence of brefeldin A (3 μg/mL) for 2 h. Cells were washed twice with PBS, fixed with 4% paraformaldehyde for 20 min at room temperature and permeabilized with permeabilization buffer (0.3% Triton X-100, 0.5% BSA, PBS) for 10 min at room temperature. Then, the cells were stained with IL-6-PE (cat. no 130-096-086) or isotype control antibody (cat. no 130-123-746) (both purchased from Miltenyi Biotec, Bergisch Gladbach, Germany) for 1 h at room temperature. After washing with permeabilization buffer, the cells were resuspended in PBS and analyzed by flow cytometry. All flow cytometry products were collected on a BD LSRFortessa (Becton Dickinson, Franklin Lakes, NJ, USA) and analyzed with FlowJo (Becton Dickinson).

### 4.4. Gene Expression Analysis

Human primary monocytes were pretreated with CPZ (1, 5, 20 µM) for 3 h and stimulated with nucleocapsid (1 µg/mL) for 48 h. RNA was isolated from cells using TRI Reagent (Molecular Research Center, Cincinnati, OH, USA) based on the manufacturer’s instructions. Next, equal amounts of RNA were reverse transcribed to cDNA using a Maxima First Strand cDNA Synthesis Kit for RT-quantitative PCR (Thermo Fisher Scientific, Waltham, MA, USA). Gene expression analysis was performed using SYBR Green I Master Mix on a LightCycler 480 (Roche, Basel, Switzerland) in a 384-well white plate. The cycling conditions were as follows: initial denaturation at 95 °C for 5 min; then 45 cycles of 95 °C for 10 s, 60 °C for 10 s, and 72 °C for 20 s. Relative mRNA levels of a cognate gene were normalized to the geometric mean of the housekeeping genes *HPRT1*, *HMBS*, and *RPL13A* as described previously [93]. The primer pair used for *IL6* was 5′-CCTGAACCTTCCAAAGATGG-3′ (forward) and 5′-GGTCAGGGGTGGTTATTGC-3′ (reverse), as previously described in Salkowska et al. [94]. The primer pair used for *IL1B* was 5′-GGACAGGATATGGAGCAACAAGTG-3′ (forward) and 5′-ACACGCAGGACAGGTACAGATTC-3′ (reverse), as previously described in Karwaciak et al. [18]. The primers for the housekeeping genes were *HPRT1*, 5′-TGACACTGGCAAAACAATGCA-3′ (forward) and 5′-GGTCCTTTTCACCAGCAAGCT-3′ (reverse); *HMBS*, 5′-GGCAATGCGGCTGCAA-3′ (forward) and 5′-GGGTACCCACGCGAATCAC-3′ (reverse); *RPL13A*, 5′-CCTGGAGGAGAAGAGGAAAGAGA-3′ (forward) and 5′-TTGAGGACCTCTGTGTATTTGTCAA-3′ (reverse), which were taken from the work of Vandesompele et al. [93].

### 4.5. ELISA for the Detection of IL-6

Cell culture supernatants from human monocytes cultured for 48 h in the presence of the nucleocapsid SARS-CoV-2 protein were analyzed using the Human IL-6 Quantikine ELISA Kit (R&D Systems, Minneapolis, MN, USA), following methods based on the manufacturer’s instructions. Absorbance at 450 nm was read in a Sunrise microplate reader (Tecan, Männedorf, Switzerland).

### 4.6. NF-κB Translocation

Cells were seeded in 96-well collagen-coated plates at a density of 2 × 10^4^ cells per well. Cells were pretreated with CPZ (20 μM) for 3 h and stimulated with nucleocapsid (1 μg/mL) for 1 h. After treatment, the cells were washed with PBS, fixed with 4% paraformaldehyde for 20 min at room temperature and permeabilized with 0.1% Triton X-100 for 5 min at room temperature. After 3 washes with PBST washing buffer (PBS, 0.1% Tween-20), the cells were stained with NF-κB p65 (D14E12) Alexa Fluor 488 conjugated antibody (Cell Signaling, Danvers, MA, USA) overnight at 4 °C. Then, the cells were washed 3 times with PBST and incubated with nuclear staining solution (2 μg/mL Hoechst 33342 in PBS) for 30 min. Fluorescence was measured using the Cellomics ArrayScan HCS Reader and image analysis software (Thermo Fisher Scientific). The Cytoplasm to Nucleus Translocation BioApplication software (Thermo Fisher Scientific) was used to calculate the difference in nuclear and cytoplasmic fluorescence intensity. Nuclear extracts were prepared using NE-PER Nuclear and Cytoplasmic Extraction Reagents (Thermo Fisher Scientific) and analyzed subsequently using Western blotting.

### 4.7. Western Blotting

Cells were pretreated with CPZ (20 μM) for 3 h and stimulated with nucleocapsid (1 μg/mL) for 30 min or for 1 h (to detect NF-κB p65 translocation). Whole cell lysates were prepared using RIPA buffer (50 mM Tris-HCl pH 8.0, 150 mM NaCl, 0.1% Triton X-100, 0.1% SDS, 0.5% sodium deoxycholate), while cytoplasmic and nuclear extracts were prepared using an NE-PER Nuclear and Cytoplasmic Extraction Kit (Thermo Fisher Scientific) according to the manufacturer’s protocol. Protein concentrations of whole cell lysates and cytoplasmic and nuclear extracts were measured by a Pierce BCA Protein Assay kit (Thermo Fisher Scientific). Proteins were electrophoresed on a 12% Bis–Tris NuPage precast gel (Thermo Fisher Scientific) and transferred to nitrocellulose membranes. The membranes were blocked using 5% nonfat milk in TBST for 1 h, followed by incubation with primary antibodies overnight at 4 °C. The following primary antibodies were used: NF-κB p65 (D14E12), phospho-IκBα (Ser32/36) (5A5), phospho-MEK1/2 (Ser217/221), MEK1/2 (D1A5), phospho-p38 MAPK (Thr180/Tyr182) (D3F9), p38 MAPK (Cell Signaling, Danvers, MA, USA), phospho-ERK (E-4), ERK1/2 (C-9) (Santa Cruz, Dallas, TX, USA), anti-beta actin, and anti-TBP (Abcam, Cambridge, UK). Next, the membranes were washed with TBST and incubated for 1 h with HRP-conjugated goat anti-rabbit secondary antibody (ab6721, Abcam) or goat anti-mouse secondary antibody (#32430, Thermo Fisher Scientific) at room temperature, and specific bands were detected using SuperSignal West Pico Chemiluminescent Substrate (Thermo Fisher Scientific). Membranes were then scanned using the G-Box chemiluminescence imaging station (Syngene, Cambridge, UK). Quantification of the western blots was performed using ImageJ software (http://imagej.nih.gov/ij/, accessed on 31 May 2022).

### 4.8. Statistics

Statistical analysis was performed using SigmaStat ver.3.5 (Systat Software Inc. San Jose, CA, USA). Data were analyzed using one-way Friedman Repeated Measures ANOVA on Ranks followed by Student-Newman–Keuls post hoc test. The significance cutoff was set at *p* < 0.05.

## 5. Conclusions

In summary, a growing amount of data, including those presented in the current manuscript, suggests that chlorpromazine may be a low-cost supportive treatment for severe cases of COVID-19. Its high bioavailability, high lung concentrations, low side effects, and low cost make it a possible alternative to expensive therapies such as remdesivir or tocilizumab, especially in lower income countries where population immunization is still very low.

## Data Availability

Not applicable.

## Supplementary Materials

The following supporting information can be downloaded at: https://www.mdpi.com/article/10.3390/molecules27123651/s1, Figure S1. The results of densitometric analysis of the western blot images shown in Figure 5B were performed using ImageJ; Figure S2. The results of densitometric analysis of the western blot images shown in Figure 6 were performed using ImageJ; Original Western blot scans.
