# Supplementary material for: Chlorpromazine, a Clinically Approved Drug, Inhibits SARS-CoV-2 Nucleocapsid-Mediated Induction of IL-6 in Human Monocytes
Source: Molecules. 2022 Jun 7;27(12):3651. doi: 10.3390/molecules27123651 (PMC9228867; doi:10.3390/molecules27123651)

Supplementary Materials

Chlorpromazine, a clinically approved drug, inhibits SARS-CoV-2 nucleocapsid-mediated induction of IL-6 in human monocytes.

Iwona Karwaciak, Kaja Karaś, Anna Sałkowska, Joanna Pastwińska and Marcin Ratajewski

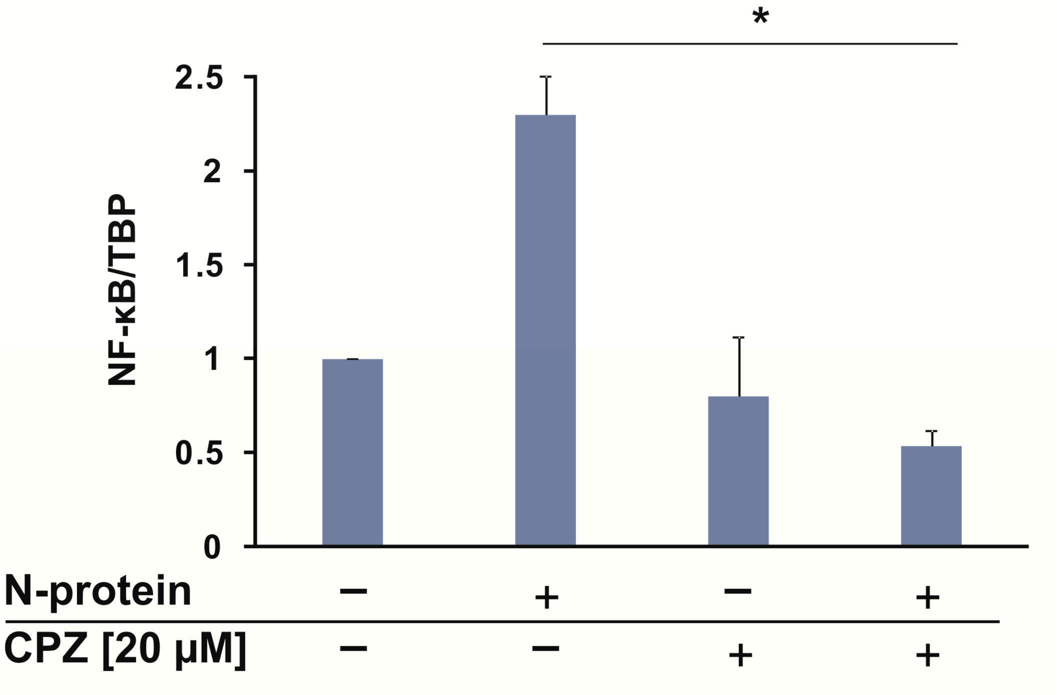

Figure S1. The results of densitometric analysis of the western blot images shown in Figure 5B were performed using ImageJ. Asterisks indicate a statistically significant difference at $p < 0.05$. Results are shown as mean +/- S.D., n=4.

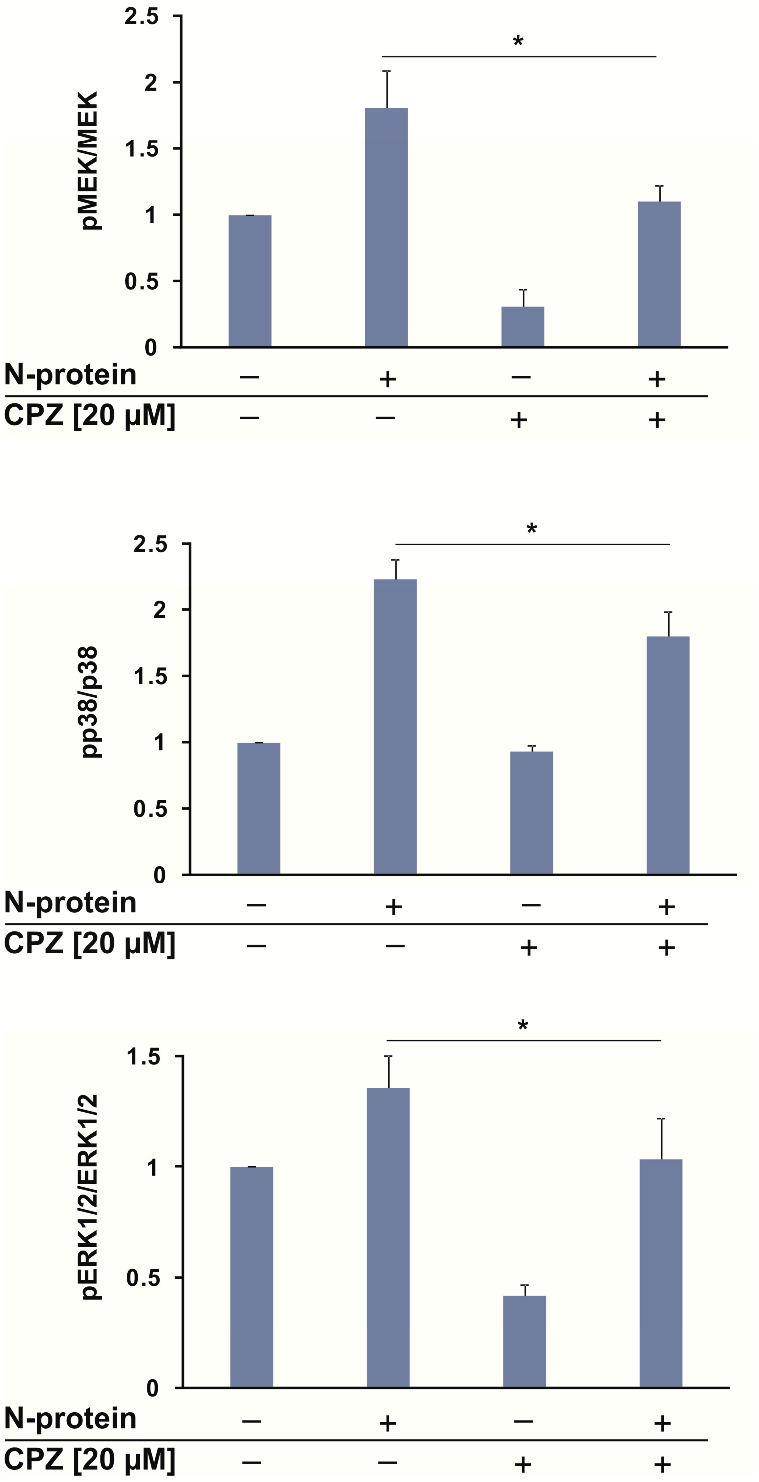

Figure S2. The results of densitometric analysis of the western blot images shown in Figure 6 were performed using ImageJ; p-protein and total protein values were normalized to the loading control (β-actin) and the results are shown as the mean +/- S.D. of phospho/total ratio of normalized values, n=4. Asterisks indicate a statistically significant difference at $p < 0.05$.

ORIGINAL SCAN BLOTS

NF-κB p65

1. Control, **2**. Nucleocapsid, **3**. Chlorpromazine, **4**. Chlorpromazine + Nucleocapsid

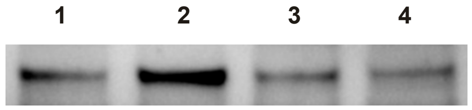

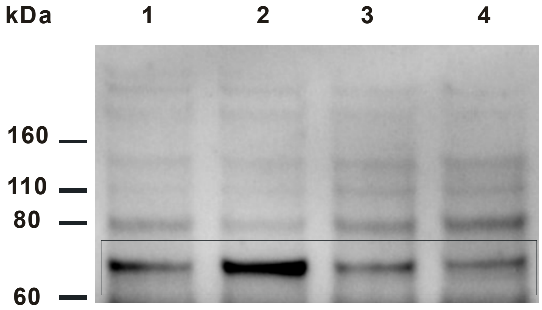

TATA binding protein TBP

1. Control, **2**. Nucleocapsid, **3**. Chlorpromazine, **4**. Chlorpromazine + Nucleocapsid

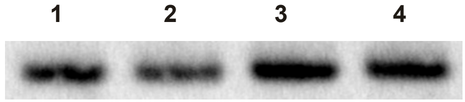

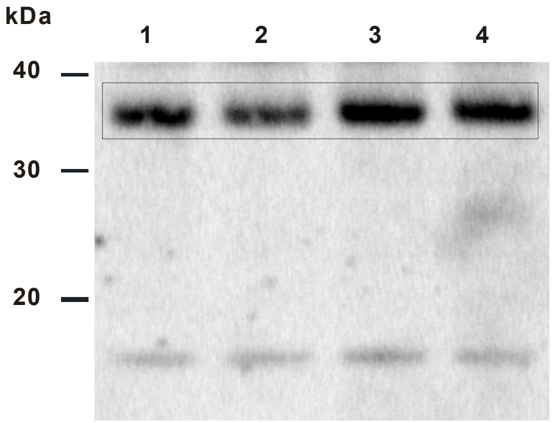

p-ERK1/2

1. Control, **2**. Nucleocapsid, **3**. Chlorpromazine, **4**. Chlorpromazine + Nucleocapsid

1 2 3 4

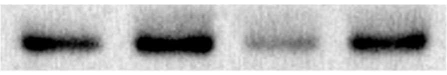

kDa 1 2 3 4

50
40
30

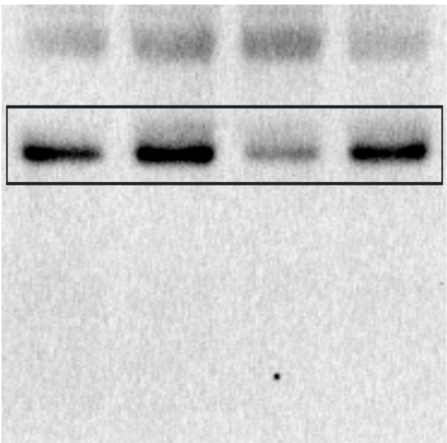

ERK1/2

1. Control, **2**. Nucleocapsid, **3**. Chlorpromazine, **4**. Chlorpromazine + Nucleocapsid

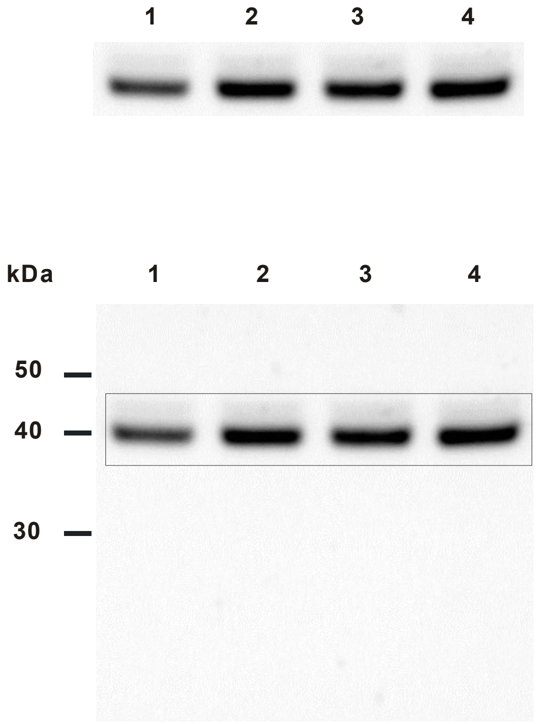

p-MEK

1. Control, **2**. Nucleocapsid, **3**. Chlorpromazine, **4**. Chlorpromazine + Nucleocapsid

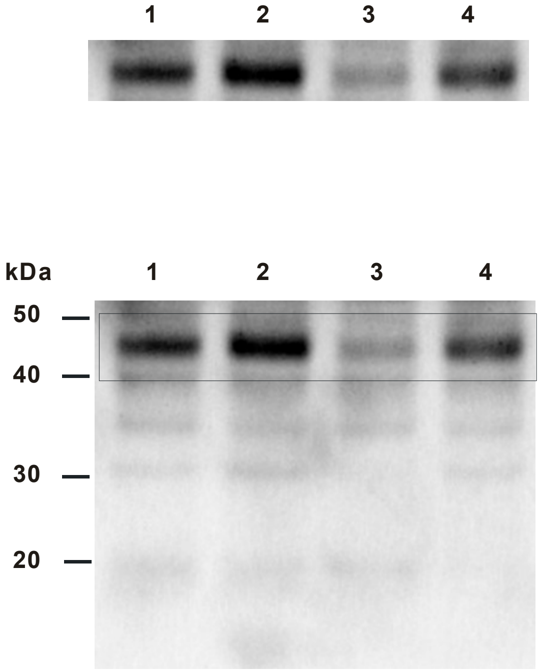

MEK

1. Control, **2**. Nucleocapsid, **3**. Chlorpromazine, **4**. Chlorpromazine + Nucleocapsid

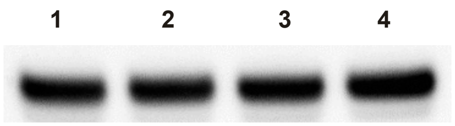

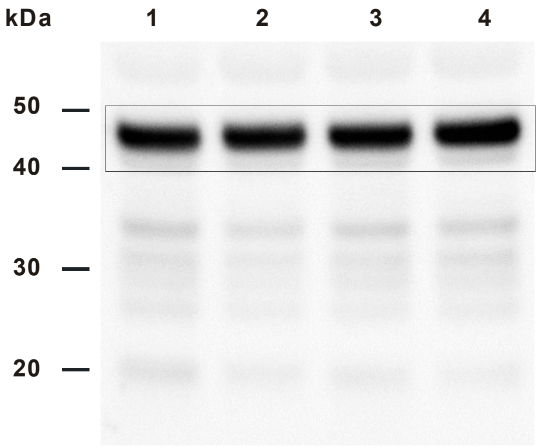

p-p38

1. Control, **2**. Nucleocapsid, **3**. Chlorpromazine, **4**. Chlorpromazine + Nucleocapsid

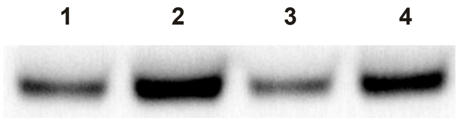

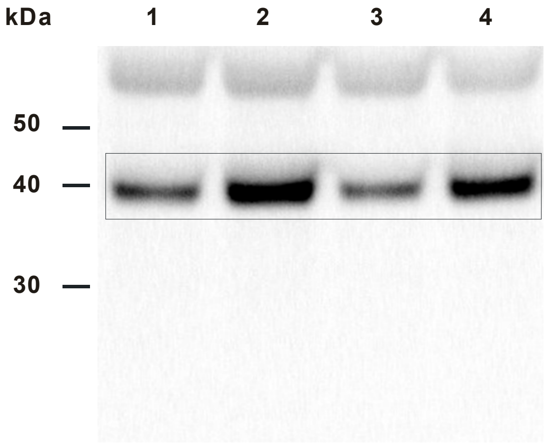

p38

1. Control, **2**. Nucleocapsid, **3**. Chlorpromazine, **4**. Chlorpromazine + Nucleocapsid

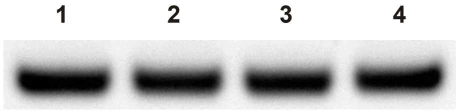
1
2
3
4

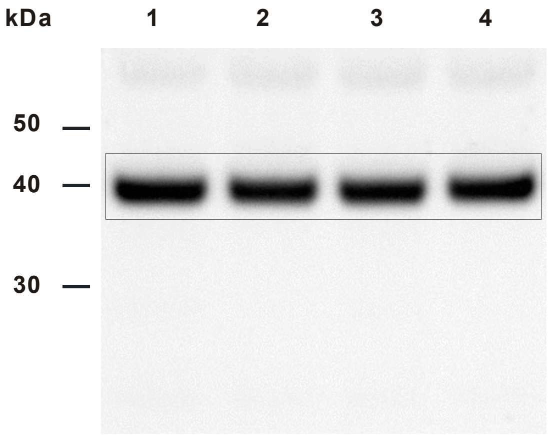
kDa
1
2
3
4
50
40
30

Beta actin

1. Control, **2**. Nucleocapsid, **3**. Chlorpromazine, **4**. Chlorpromazine + Nucleocapsid

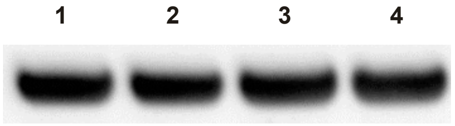

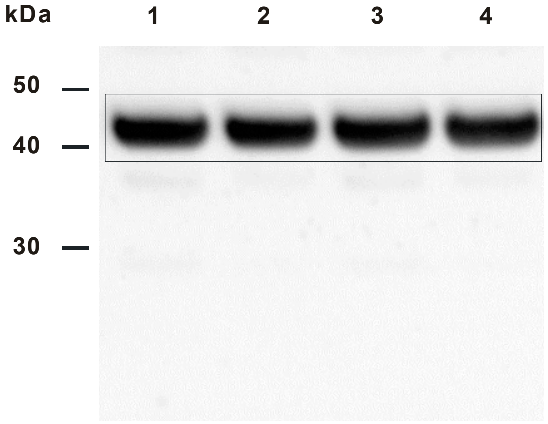

Supplement: Supplementary file 1 [file molecules-27-03651-s001.zip › molecules-1724391-supplementary.pdf]
